# Macroscopic Biaxial Order in Multilayer Films of Bent-Core Liquid Crystals Deposited by Combined Langmuir–Blodgett/Langmuir–Schaefer Technique

**DOI:** 10.3390/nano14040357

**Published:** 2024-02-14

**Authors:** Francesco Vita, Fabrizio Corrado Adamo, Mario Campana, Blake Bordokas, Federica Ciuchi, Maria Penelope De Santo, Daniel Hermida-Merino, Angela Lisovsky, Michela Pisani, Diego Pontoni, Eric Scharrer, Oriano Francescangeli

**Affiliations:** 1 Department of Science and Engineering of Matter, Environment and Urban Planning (SIMAU), Polytechnic University of Marche, Via Brecce Bianche, 60131 Ancona, Italy; f.c.adamo@staff.univpm.it (F.C.A.); m.pisani@univpm.it (M.P.); 2 ISIS Neutron and Muon Source, Rutherford Appleton Laboratory, Harwell Campus, Didcot OX11 0QX, UK; mario.campana@stfc.ac.uk; 3 Department of Chemistry, University of Puget Sound, Tacoma, WA 98416, USA; blake@bordokas.com (B.B.); angelalisovsky@outlook.com (A.L.); escharrer@pugetsound.edu (E.S.); 4 CNR-Nanotec c/o Physics Department, University of Calabria, Ponte Bucci, Cubo 31C, 87036 Arcavacata di Rende, Italy; federica.ciuchi@cnr.it (F.C.); maria.desanto@fis.unical.it (M.P.D.S.); 5 Physics Department, University of Calabria, Ponte Bucci, Cubo 31C, 87036 Arcavacata di Rende, Italy; 6 DUBBLE@ESRF BP CS40220, 38043 Grenoble, France; daniel.hermida_merino@esrf.fr; 7 Departamento de Física Aplicada, Centro de Investigación en Nanomateriais e Biomedicina (CINBIO), Universidade de Vigo, Campus Lagoas-Marcosende, E36310 Vigo, Spain; 8 ESRF—The European Synchrotron, 71 Avenue des Martyrs, 38043 Grenoble, France; diego.pontoni@esrf.fr

**Keywords:** liquid crystals, bent-core mesogens, Langmuir films, grazing-incidence wide-angle X-ray scattering, neutron reflectivity, OC4-2MePh(mono2MeODBP)

## Abstract

Bent-core liquid crystals, a class of mesogenic compounds with non-linear molecular structures, are well known for their unconventional mesophases, characterized by complex molecular (and supramolecular) ordering and often featuring biaxial and polar properties. In the nematic phase, their unique behavior is manifested in the formation of nano-sized biaxial clusters of layered molecules (cybotactic groups). While this prompted their consideration in the quest for nematic biaxiality, experimental evidence indicates that the cybotactic order is only short-ranged and that the nematic phase is macroscopically uniaxial. By combining atomic force microscopy, neutron reflectivity and wide-angle grazing-incidence X-ray scattering, here, we demonstrate that multilayer films of a bent-core nematic, deposited on silicon by a combined Langmuir–Blodgett and Langmuir–Schaefer approach, exhibit macroscopic in-plane ordering, with the long molecular axis tilted with respect to the sample surface and the short molecular axis (i.e., the apex bisector) aligned along the film compression direction. We thus propose the use of Langmuir films as an effective way to study and control the complex anchoring properties of bent-core liquid crystals.

## 1. Introduction

**Figure 1:**
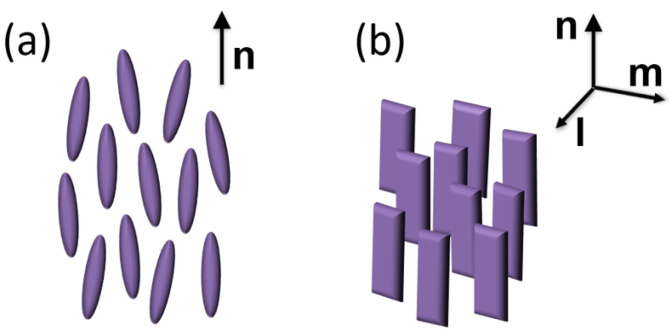
Schematic representation of (**a**) uniaxial N phase of rod-like molecules; (**b**) biaxial N phase of board-like molecules. **n**, **m** and **l** are the molecular directors.

Among the several different mesophases formed by liquid crystalline materials, the nematic (N) phase of calamitic (i.e., rod-like) liquid crystals (LCs) is by far the most well known, as it provides the basis for the widespread LC display technology. Its fundamental feature is the spontaneous alignment of the molecules’ long axes along a common average direction, denoted by the molecular director **n** (Figure 1a). As a consequence, all the physical properties of a N material are strongly anisotropic, with characteristic uniaxial symmetry.

For the N phase to be of any practical utility, it is necessary to control the orientation of the **n** director over macroscopic distances. In typical LC devices, this task is accomplished by imposing anisotropic surface anchoring conditions at the confining boundaries, which forces **n** to align along a predetermined preferential direction; once the ground state orientation of **n** has been established, it can then be switched through the torque exerted by an external electric field.

Actually, theoretical considerations do not forbid the possibility of a biaxial N phase, where the average orientation of the long molecular axis along **n** is accompanied by the preferential alignment of a molecular transverse axis along a secondary director **m** orthogonal to **n** (Figure 1b) [1,2]. However, despite considerable experimental effort, the biaxial N phase has remained elusive, with no undisputable evidence reported until now for low-molecular-weight calamitic LCs [3,4]. Over the last two decades, bent-core mesogens (BCMs), a class of compounds featuring a kinked aromatic core between two terminal aliphatic tails, have attracted considerable interest for the formation of a variety of exotic mesophases and self-assembled supramolecular structures [5,6,7,8], as well as for the exhibition of a possibly biaxial N phase [5,9,10,11]. Although the occurrence of spontaneous macroscopic biaxiality in nematic BCMs has been questioned, strong experimental evidence suggests the presence of local biaxial (and possibly polar) order within nanosized clusters of stratified molecules (known as cybotactic groups) which permeate the N phase. While the clusters’ transverse axes are randomly oriented in the unperturbed N phase, resulting in an overall uniaxial mesophase, proper external stimuli can align the clusters, extending biaxial (and possibly polar) order over a macroscopic length scale [9,10,11,12,13,14].

A critical issue for the experimental study of BCMs, as well as for their use in electro-optical devices, is the capability to finely control the orientation of the molecular director by means of proper anchoring conditions. Unfortunately, the surface treatments known to be effective for aligning conventional nematics often provide unexpected results when used with BCMs [15,16,17]. Notably, this ambiguity in the alignment of BCMs has resulted in conflicting interpretations of several experiments, e.g., with claims of N biaxiality being reinterpreted in terms of anchoring transitions in a uniaxial N phase [18,19,20]. Clearly, the experimental demonstration of N biaxiality in any LC system would be extremely eased by the ability to align both **n** and **m** directors by means of biaxial anchoring conditions.

An unconventional strategy to tackle the BCM alignment problem is the deposition of BCM Langmuir films: mesogens spread over water form a thin layer at the water–air interface, with molecular packing controlled by the pressure exerted on the film by a movable barrier; once the desired molecular arrangement has been obtained, the film can be transferred onto a solid substrate. This technique, already used to deposit rod-like materials of different natures like cellulose nanocrystals [21] or carbon nanotubes [22], represents a valuable tool to determine the surface alignment of BCMs and to study their anchoring properties. The resulting films are of interest by themselves, e.g., in the case of ferroelectric LCs, or they can be used as aligning substrates for bulk LCs [23,24,25,26,27,28,29,30,31,32,33,34,35,36,37,38].

**Figure 2:**
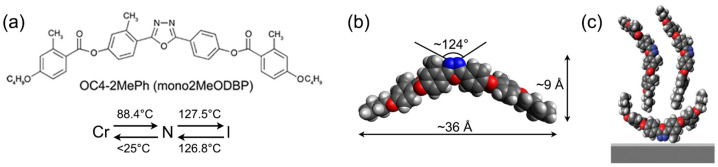
(**a**) Chemical structure of mesogen OC4-2MePh(mono2MeODBP) with the relative phase transition temperatures on heating and cooling: crystalline phase (Cr)–nematic phase (N)–isotropic phase (I). (**b**) Optimized molecular geometry. (**c**) Structure of a Langmuir film deposited on a silicon substrate (with a thin silicon oxide layer at the interface), according to [38].

Recently, we have used this approach to deposit thin films of a BCM known as OC4-2MePh(mono2MeODBP) [38]. It belongs to a widely studied family of laterally methylated BCMs featuring an oxadiazole bisphenol core and short butoxy terminal chains (Figure 2a,b) [39,40,41,42]. The N phase of these mesogens exhibits a few peculiarities: the possibility to be supercooled to room temperature and the strong evidence (from X-ray diffraction data) of locally biaxial molecular ordering [40,41,42]. The formation of stable Langmuir films in these BCMs is remarkable, as their chemical structure, with two hydrophobic tails and a more hydrophilic core, is significantly different from that of typical amphiphilic molecules. In fact, the complex BCM interaction with the water sub-phase in Langmuir films is responsible for the unusual bilayer structure of deposited films revealed by X-ray reflectivity (XRR) measurements: an upper layer of upright molecules and a bottom layer of flat molecules (Figure 2c) [38].

While XRR can elucidate the film structure in the direction orthogonal to the substrate, it is blind to in-plane order. The latter can be conveniently investigated by means of grazing-incidence wide-angle X-ray scattering (GIWAXS). Unfortunately, because of their chemical structure (lacking heavy atoms) and short-range positional order, LCs exhibit weak and diffuse diffraction features. As a result, films obtained with a single deposition are too thin to generate a detectable diffraction signal. To address this problem, here, we describe the preparation of multilayer samples via repeated Langmuir–Blodgett (LB)/Langmuir–Schaefer (LS) deposition. GIWAXS measurements performed on these samples reveal a tilted, smectic-C like, molecular stratification and macroscopic in-plane ordering; the films are thus biaxial. The structure of deposited films is compared with that of Langmuir films over water, investigated by means of neutron reflectivity (NR). The results provide further insight into the BCM assembly at the air–water interface, showing that molecular ordering is substantially preserved during the deposition process.

## 2. Materials and Methods

### 2.1. Mesogen Synthesis

The synthesis of OC4-2MePh(mono2MeODBP) has been described elsewhere [39]. Its chemical structure, typical dimensions in the fully extended configuration and mesophase diagram are shown in Figure 2a,b.

### 2.2. Sample Preparation

Langmuir films of OC4-2MePh(mono2MeODBP) over water were prepared in a NIMA Langmuir–Pockels trough equipped with control barriers (NIMA Technology Ltd., Coventry, UK). A 0.1 mg/mL solution of OC4-2MePh(mono2MeODBP) in chloroform was spread over pure water (resistivity of 18.2 MΩ/cm); the latter was obtained from an ELGA PURELAB Flex water purification system (ELGA LabWater, Wycombe, UK). After the evaporation of the solvent, the film was compressed by closing the control barriers at a constant rate of 70 cm^2^/min from an initial area per molecule of *A* ≈ 130 Å^2^. Compression isotherms (surface pressure Π vs. area per molecule *APM*) were measured using a Wilhelmy plate pressure sensor.

**Figure 3:**
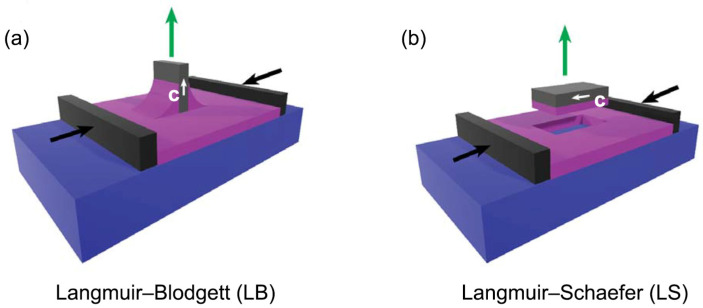
Schematic drawing of the deposition set-up, with the Langmuir film (purple) being transferred from the water (blue) onto the silicon substrate (gray): (**a**) LB technique used to transfer the first layer; (**b**) LS technique used to deposit the subsequent 28 layers. The orientation of the substrate during the LB and LS deposition phases is indicated by the in-plane vector **c** (white arrow).

Multilayer samples were obtained by repeated film depositions on silicon substrates ((111)-cut *p*-doped), previously treated with piranha solution. Using (111)-cut silicon prevented the appearance of Bragg reflections in the *q*-range of interest for GIWAXS experiments. The deposition pressure was set at Π = 30 mN/m, corresponding to an area per molecule of *APM* ≈ 32 Å^2^. The deposition of the first layer was performed with the LB technique, i.e., by extraction of a vertical substrate immersed in the water before the formation of the Langmuir film (Figure 3a). Subsequently, 28 additional layers (for a total of 29 layers) were deposited using the LS configuration (Figure 3b): in this case, the substrate, kept parallel to the water surface, was gently lowered until it came in contact with the Langmuir film, which was hence transferred onto the silicon. In the LS depositions, the substrate orientation was chosen in such a way that the barrier compression direction was parallel to the substrate extraction direction of the first LB deposition. We denote this direction by the in-plane vector **c**. After each layer deposition, the samples were thoroughly dried by a nitrogen flux.

### 2.3. Atomic Force Microscopy Measurements

Samples obtained by a single LB deposition on silicon were investigated by atomic force microscopy (AFM) using a Multimode 8 AFM microscope equipped with a Nanoscope V controller (Bruker, Santa Barbara, CA, USA). Data were acquired in tapping mode using silicon cantilevers (model TAP150, Bruker, Santa Barbara, CA, USA).

### 2.4. Neutron Reflectivity Measurements

NR measurements on Langmuir films were performed at the Surf beamline of ISIS Neutron and Muon Source (Rutherford Appleton Laboratory, Didcot, United Kingdom) [43] using a polychromatic neutron beam with wavelength *λ* between 0.5 and 7 Å. Measurements were performed at three different angles (*θ* = 0.35°, 0.65° and 1.5°) to cover a suitable *q* range ($q=\frac{4\pi \sin\theta }{\lambda }$).

Measurements were performed in two different contrasts (sub-phases) to ensure maximum sensitivity to the layer adsorbed at the interface. While the use of null-reflecting water (NRW) is particularly sensitive to the adsorbed amount at the interface, the use of D_2_O enables the resolution of the interfacial structure.

Langmuir films of OC4-2MePh(mono2MeODBP) were prepared using a NIMA trough with an area of 20 × 30 cm^2^ (NIMA Technology Ltd., Coventry, UK). A 0.1 mg/mL solution of OC4-2MePh(mono2MeODBP) in chloroform was spread on each sub-phase. Measurements were taken at a fixed surface pressure of 30 mN/m. The compression isotherms measured in this condition did not significantly differ from those measured during the previously described sample preparation experiments. In particular, the area per molecule calculated from the trough area at 30 mN/m was ~36 Å^2^, very close to the value of ~32 Å^2^ measured in deposition experiments.

The two contrasts were co-fitted using the in-house built software Rascal (version 2019), which employs the Abeles matrix formalism to calculate the neutron reflectivity from stratified media [44]. A Bayesian analysis of the fit was then used to associate an appropriate confidence interval to the optimal fitted values. The reflectivity profiles were co-fitted, modeling the air–water interface as a finite stack of layers, with each layer being characterized by a thickness *t*, an interlayer roughness *σ* and a scattering length density *Nb_layer_*. The scattering length density of a layer is as follows:

(1) $${Nb}_{layer}=\sum_{i}N_{i}b_{i},$$

where *N_i_* and *b_i_* are the number density and the scattering length for the *i*-th species present in the layer, respectively. The latter can be calculated from composition for the BCM, D_2_O and H_2_O: *b_BCM_* = 169 fm molec^−1^, *b_D2O_* = 19.1 fm molec^−1^ and *b_H2O_* = −1.67 fm molec^−1^ (note how mixing ~8% of D_2_O and 92% of H_2_O leads to NRW with *b_NRW_* = 0). D_2_O often presents some H_2_O contamination, and its *Nb* may be lower than the theoretical value of 6.35 × 10^−6^ Å^−2^. The D_2_O used in this study had an *Nb* = 6.21 × 10^−6^ Å^−2^, as calculated from the observed critical edge *q_c_* ($q_{c}=\sqrt{16\pi \Delta Nb}$, where Δ*Nb* is the difference in *Nb* between the two bulk phases, air and D_2_O).

When using NRW, the reflectivity is solely originating from the interfacial monolayer. Under these conditions, the adsorbed amount per unit area *Γ* and the corresponding area per molecule *APM* can be calculated from the experimentally determined parameters *Nb_layer_* and *t* as follows:

(2) $$\Gamma =\frac{{Nb}_{layer}t}{b_{BCM}N_{A}},$$

where *N_A_* is Avogadro’s number [45]. The *APM* is then calculated as follows:

(3) $$APM=\frac{1}{N_{A}\Gamma }.$$

The fitting of the experimental data, performed via scripting in Rascal, used the thickness *t*, the *APM* and, where applicable, the percentage of hydration water as fitting parameters for each layer. No roughness was required to fit the reflectivity data. We set the interlayer roughness at 0.5 Å in order to obtain smoother transitions in the *Nb* profiles, hence a better visualization of the plots.

### 2.5. Grazing-Incidence Wide-Angle X-ray Scattering Measurements

**Figure 4:**
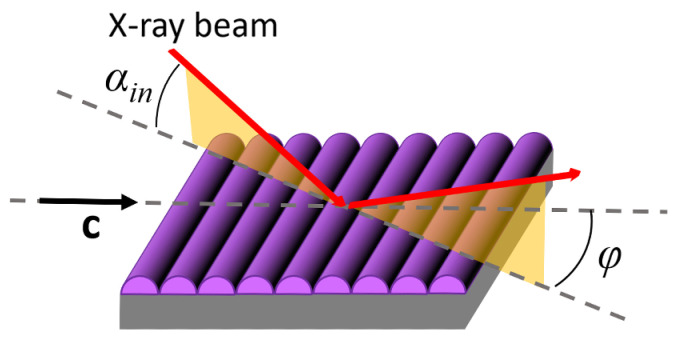
GIWAXS geometry. The drawing highlights the presence of film undulations (purple) along the **c** vector, as discussed in Section 3.1 and Section 3.3.

The in-plane and out-of-plane order of multilayer films on silicon was investigated by GIWAXS. The measurements were carried out at the BM26B-Dubble beamline of the European Synchrotron Radiation Facility (ESRF, Grenoble, France) and at the NCD-SWEET beamline of the ALBA synchrotron (Barcelona, Spain). The data here reported were collected under the following experimental conditions: the samples were mounted on a rotating stage allowing measurements under different azimuthal orientations (the *φ* angle between the beam incidence plane and the film compression direction **c**, as shown in Figure 4); the beam energy was 8.00 keV (wavelength *λ* = 1.55 Å) and the beam size was 111 × 12 μm^2^ (width × height); the sample-to-detector distance was *D* = 198 mm and the fixed incident angle was set at *α_in_* = 0.16°, leading to an irradiated area on the sample of ~0.5 mm^2^; all the patterns were recorded at room temperature, with an exposure time of 30 s, using an LX255-HS detector (Rayonix, Evanston, IL, USA).

## 3. Results and Discussion

### 3.1. Preliminary Langmuir Film Characterization

**Figure 5:**
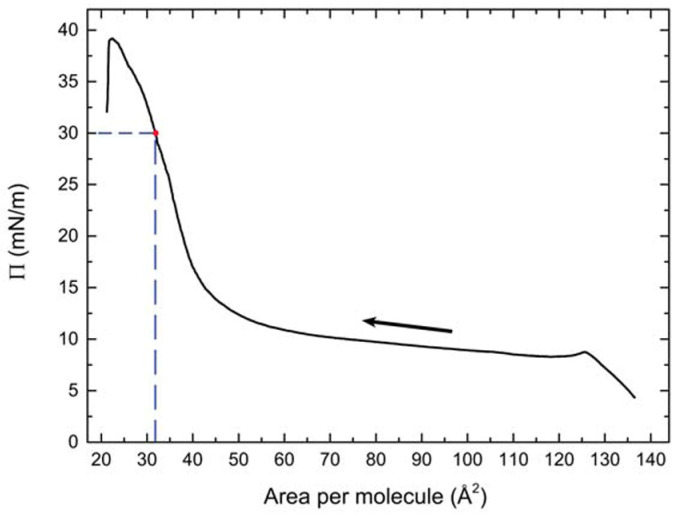
Compression isotherm of the BCM Langmuir film. The arrow indicates the compression direction; the dashed lines point out the deposition parameters.

**Figure 6:**
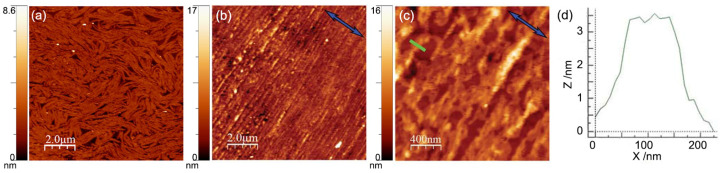
(**a**) AFM image of a single LB deposition on silicon at Π = 12 mN/m, from [38]. (**b**,**c**) AFM images of a single LB deposition on silicon at Π = 30 mN/m at different magnifications. The blue arrows indicate the film compression direction **c**. Color-coded scale bars indicate the depth of the film. (**d**) Thickness profile measured along the green bar in (**c**).

A typical compression isotherm of OC4-2MePh(mono2MeODBP) over water is shown in Figure 5. The sudden pressure drop at the end of the compression curve indicates the collapse of the film occurring at Π ≈ 39 mN/m. Below this threshold, the isotherm is characterized by a plateau at the pressure of ~10 mN/m, during which, the area per molecule decreases from ~120 to ~60 Å^2^ with only a minimal increase in the surface pressure, followed by a steep increase in the pressure. In our previous work [38], we demonstrated that the plateau is due to the coalescence and internal reorganization of the initially floating monolayer domains, with the final formation of a more stable and uniform double molecular layer in correspondence of the pressure increase. Consequently, in that case, we chose the end of the plateau, at a pressure of 12 mN/m, as the set point for the film deposition. However, the resulting film morphology was still not very homogenous, with AFM images showing the formation of fibrous supramolecular structures locally aligned with each other but without long-range orientational order (Figure 6a).

Aiming to obtain more uniform coverage of the substrate and, above all, more ordered packing of the molecules, for the present work, we chose a higher deposition pressure of 30 mN/m, corresponding to an area per molecule of ~32 Å^2^. To investigate the effect of the large deposition pressure on the film morphology, a single LB deposition on silicon was examined by AFM (Figure 6b–d). The difference with films deposited at Π = 12 mN/m is evident. The film became much more uniform; the fibrous meandering structure present in low-pressure samples was replaced by a dense sequence of parallel grooves, homogenously aligned along the direction perpendicular to the dipping direction **c** (Figure 6b,c). These undulations had widths of ~100 nm and depths of ~3 nm (Figure 6d), values quite similar to those observed in samples deposited at lower pressure. Clearly, the higher deposition pressure resulted in a more ordered mesoscale structure, characterized by an in-plane anisotropy extending uniformly over the sample surface.

### 3.2. Neutron Reflectivity Measurements

**Figure 7:**
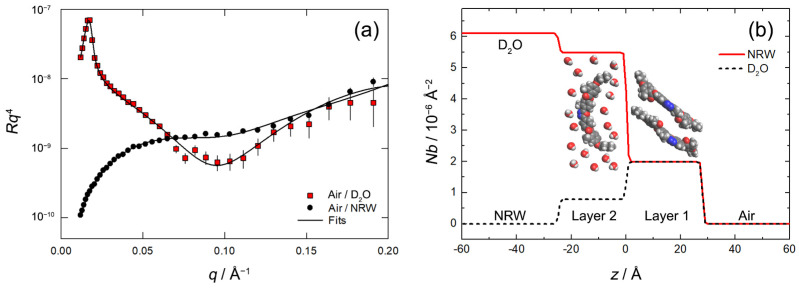
(**a**) Reflectivity profiles for the BCM layer at the air–water interface, plotted as *Rq*^4^ vs. *q*, with *R* indicating the reflectivity. Red squares represent the data for the air/D_2_O interface; black circles correspond to the air/NRW interface. The solid lines represent the best fit to the data. (**b**) Scattering length density profiles for the BCM layer at the air–water interface. Red and black solid lines correspond to D_2_O and NRW runs, respectively. The zero of the *z* axis was arbitrarily positioned at the interface between Layer 1 and Layer 2, which represents the boundary between air and water (Layer 1 is entirely out of the water phase; Layer 2 is immersed in the bulk). A schematic representation of the interfacial bilayer is superimposed on the plot.

To further investigate the effect of the increased deposition pressure on the film morphology, NR measurements were performed on the BCM Langmuir film at a fixed pressure of 30 mN/m, representing conditions prior to deposition. Figure 7a shows the reflectivity curves measured on D_2_O and NWR along with the best line fits; the corresponding *Nb* profiles are shown in Figure 7b together with a molecular model of the interfacial layer. Attempts to fit the reflectivity profiles through a one-layer model proved unsuccessful. Satisfactory results were obtained with the addition of a second layer. The fit results indicate that the upper layer (Layer 1) contains only BCM molecules (no hydration): this is often observed within Langmuir monolayers where the hydrophobic part is expelled out of the aqueous phase and the Van der Waals interactions within the monolayer ensure tight packing. The lower layer (Layer 2) consists of BCM and water and represents a diffuse layer on the aqueous side of the interface. It must be stressed that modeling this diffuse layer on the air side of the interface (i.e., as a layer composed of air and BCM molecules) led to an unsatisfactory fit of the data.

The fitted parameters are summarized in Table 1 with the corresponding 65% confidence intervals. The thickness of the upper layer (27.8 Å) is significantly lower than the extended full length of the molecule (~36 Å) and is consistent with an emerged layer of tilted BCM molecules (Figure 7b). In fact, the measured value is very close to the layer thickness provided by GIWAXS for deposited multilayers (25.1 Å), wherein molecules assume a tilted configuration (as discussed in the next section). The *APM* within this layer is 30.5 Å^2^, indicating that the BCM molecules are very well-ordered within the monolayer. This small *APM* value is evidently incompatible with the molecules lying flat. Also, in this case, the *APM* is very close to the value estimated by GIWAXS for deposited multilayers (33.9 Å^2^; see next section), further supporting the conclusion that the tilted molecular arrangement observed in deposited films is already present in the Langmuir film prior to deposition.

The thickness of the bottom layer is slightly smaller, but it is highly hydrated (77% water in the layer) and contains considerably less material (28% of the overall interfacial BCM content). The latter percentage is similar to the results of previous XRR measurements on deposited films, which revealed a ratio of 1.92: 1 between the number of molecules in the top and in the bottom layer [38]. The larger value of *APM* for Layer 2 (77.5 Å^2^, quite close to the value of 69.1 Å^2^ obtained from XRR measurements in anhydrous conditions) would suggest an arrangement with the molecules lying flat with lifted terminal tails. However, the high level of hydration and the layer thickness, large for a layer of flat molecules, rather indicate a highly dispersed layer of relatively disordered molecules.

In evaluating the details of the film structure, one should also consider that our two-layer model necessarily provides a simplified picture, as it does not take into account the variation in *Nb* across the BCM molecules (core vs. tails) and the possibility of a partial layer intermixing (e.g., by tail interdigitation) which would make the boundary between the two layers somewhat ill defined. Actually, the total thickness of the interfacial film is 52.8 Å, a value very close to that of 49 Å obtained by XRR for dry Langmuir films deposited on silicon at a pressure of 12 mN/m [38]. Considering the different deposition pressure and the different content of water, the results of the two techniques are in substantial agreement in describing the system.

### 3.3. Grazing-Incidence Wide-Angle X-ray Scattering Measurements

**Figure 8:**
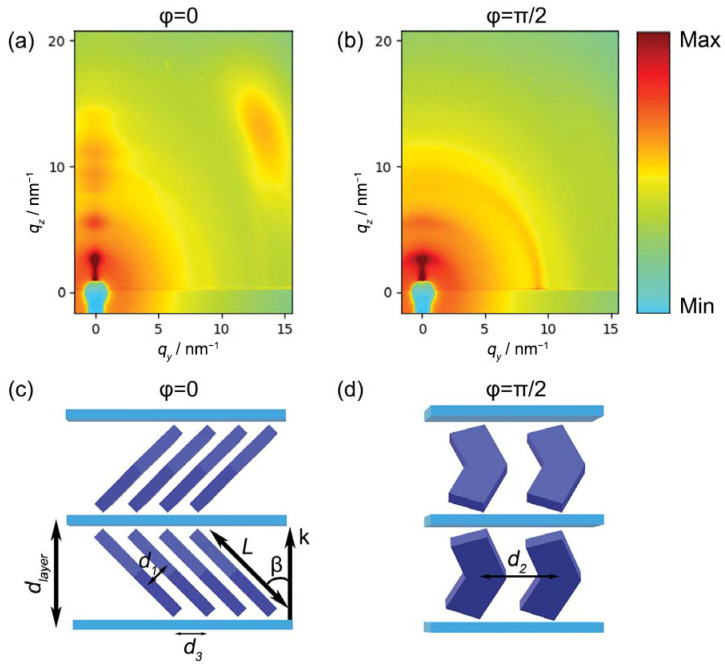
GIWAXS diffraction patterns for different azimuthal orientations of the sample: (**a**) incidence plane of the X-ray beam parallel to the film compression direction **c** (*φ* = 0); (**b**) incidence plane perpendicular to the film compression direction **c** (*φ* = *π*/2). Only the right halves of the patterns are shown because of the pattern symmetry. The diffraction intensity is color-coded on a logarithmic scale. On the lower panel, scheme of the layered molecular arrangement for different azimuthal orientations: (**c**) *φ* = 0 and (**d**) *φ* = *π*/2.

The multilayer films deposited on silicon with the combined LB/LS methodology described in Section 2.2 were characterized by GIWAXS with the geometry shown in Figure 4. Typical diffraction patterns measured for *φ* = 0 (incidence plane parallel to **c**) and *φ* = *π*/2 (incidence plane perpendicular to **c**) are shown in Figure 8a,b, respectively. The difference between the two diffraction patterns is a clear indication of the sample in-plane anisotropy, already evidenced by the AFM scans. However, while the anisotropy observed by AFM is relative to the mesoscale sample morphology, the GIWAXS data pertain to a much lower length scale, revealing the anisotropy of the molecular ordering.

Looking into the details of the GIWAXS patterns, it is possible to notice a sequence of reflections centered at *q_y_* = 0. They consist of a fundamental reflection at *q_z_* = 2.51 nm^−^^1^ and the corresponding higher orders. These reflections, present in both patterns, are clearly related to the multilayer nature of the sample and correspond to an interlayer distance *d_layer_* = 25.1 Å. This value is significantly lower than the molecular length (*L* = ~36 Å). Assuming no intercalation, this suggests a tilt of the molecules, with the molecular long axis forming an angle *β* = cos^−1^(*d_layer_/L*) = 46° with the layer normal (Figure 8c).

Comparing this sequence of reflections in the two patterns, it can be noticed that they are much more intense and transversally narrow in the pattern taken at *φ* = 0 (Figure 8a). By contrast, a significant transverse broadening affects the reflections in the patterns taken at *φ* = *π*/2 (Figure 8b), which also smears out the diffraction intensity. This effect is attributed the presence of grooves similar to those evidenced by AFM scans for single LB depositions (Figure 6b,d), i.e., to undulations of the layers propagating along the Langmuir film compression direction **c**, as shown in Figure 4.

Figure 8a also shows a broad oblique reflection in the wide-angle region, approximately centered at (*q_y_* ≈ 13.3 nm^−^^1^, *q_z_* ≈ 12.7 nm^−^^1^). The same feature is absent in the pattern taken at *φ* = *π*/2. The **q** vector of this reflection forms an angle of ~46° with the layer normal; this value exactly matches the angle *β* estimated above and corresponds to an intermolecular distance *d*_1_ ≈ 3.42 Å, the typical value of the intermolecular distance between stacked aromatic groups. Based on these observations, we attribute the wide-angle reflection to the transverse (face-to-face) positional correlation between close-packed mesogens, tilted with respect to the layer normal, as schematically shown in Figure 8c. As is typical in fluid LC systems, this transverse correlation is very short-ranged, as indicated by the breadth of the corresponding reflection. Finally, the disappearance of this oblique reflection for *φ* = *π*/2 indicates that the molecules tilt in the plane orthogonal to the compression direction **c**. Although the diffraction patterns at *φ* = 0 (Figure 8a) only show one wide-angle reflection (because of the detector being off-center), a rotation of the sample by *π* results in the same diffraction pattern. This symmetry implies an equivalent number of molecules tilted to the right and to the left of the **c** direction. This could be due, for example, to the presence of domains with opposite tilt as well as to layers with alternate tilt directions, as shown in the model of Figure 8c.

For *φ* = *π*/2, the wide-angle diffraction feature is substituted by an in-plane peak centered at (*q_y_* = 9.12 nm^−^^1^, *q_z_* = 0 nm^−^^1^). It corresponds to a *d*-spacing *d*_2_ = 6.89 Å, which can be interpreted as the transverse intermolecular distance measured in the plane of the mesogen cores (Figure 8d). While the transverse broadening of this reflection may be once again attributed to the film undulations, this diffraction feature is rather narrow in the radial direction (along *q_y_*). This indicates a relatively long-ranged positional order in the plane of the film along the compression direction. The layer area associated with each molecule can be calculated as the product of *d*_2_ and $d_{3}=\frac{d_{1}}{\cos\beta }=4.92\;Å$ (see Figure 8c,d), obtaining a value of 33.9 Å^2^, in excellent agreement with the *APM* value estimated by NR measurement on the water subphase.

## 4. Conclusions

The NR measurements performed on Langmuir films substantially confirm the complex nature of BCM assembly over water, with the mesogens organizing in a double molecular layer, most likely to decrease the interaction with water of the hydrophobic terminal chains. The top layer is tightly packed and lies entirely above the water surface, whereas the bottom layer is more diffuse and totally submerged. Transferring the Langmuir film onto a solid substrate via LB deposition preserves this double-layer structure (with the bottom layer assuming a more ordered configuration upon water evaporation), as formerly demonstrated [38]. Here, we showed that subsequent depositions via LS methodology result in biaxial films made of a stack of titled molecules with in-plane anisotropy. The layer thickness and the *APM* measured in the deposited films are very close to the corresponding values in the top layer of the Langmuir films, indicating that the tilted molecular ordering is already present at the water–air interface and is preserved in the deposition process. Apparently, after the first LB deposition, the more diffuse bottom layer of the Langmuir film does not take part in the subsequent LS depositions and does not affect the final film structure.

A remarkable feature of the deposited films is their in-plane anisotropy: considering the large investigated area in the GIWAXS geometry, it extends homogenously over macroscopic distances. This peculiar arrangement is clearly related to the inherent anisotropy of the sample preparation method, with the compression direction breaking the in-plane symmetry of the Langmuir film. It is interesting to observe that the in-plane anisotropy (and hence the film biaxiality) manifests itself at several levels: on the mesoscale, in the formation of grooves uniformly aligned along the **c** direction; on the molecular length scale, in the tilt of the long molecular axis in the plane orthogonal to **c** and in the coherent alignment of the molecular transverse axes (proper biaxiality). The last effect is not entirely new for OC4-2MePh(mono2MeODBP), as well as for other laterally substituted oxadiazole-based compounds, with X-ray diffraction measurements evidencing a strong tendency to biaxial packing in the N phase of this class of mesogens [40,41,42]. However, in the bulk, such ordering averages out over macroscopic distances, resulting in a uniaxial N phase, as proved by a thorough optical investigation [20]. The described deposition technique represents, hence, an effective strategy to extend the peculiar tendency toward local biaxial ordering of this BCM, forcing the mesogens to adopt a uniform organization over a large area.

Finally, it is worth noting that the phase diagram of bulk OC4-2MePh(mono2MeODBP) does not show any smectic mesophase. The tilted stratified structure of our film, resembling a smectic C phase, is thus artificially created by the fabrication method (although it is surprisingly stable, being observed several weeks after the film deposition). The SmC phase of BCMs is interesting as it can feature polar (ferroelectric or antiferroelectric) properties, like in the well-known SmCP (or B_2_) phase, through the coherent alignment of the transverse molecular dipoles (pointed along the apex bisectors) [8,27,28]. In our case, we demonstrated that the molecules are all tilted in the same plane, perpendicular to the film compression direction **c**, and that the molecular dipoles are perpendicular to this plane, and hence parallel to **c** (Figure 8c,d). This is remarkable, as it is a prerequisite to exhibit a SmCP-like polarization. However, the polar character of the film could not be definitely proved by our measurements as GIWAXS is unable to distinguish between opposite orientations of the apex bisectors, and hence of the molecular dipoles. This aspect certainly deserves further investigation through electro-optical characterization. Another interesting question to be elucidated is whether the mesoscale undulations observed in our films could be caused by a frustration mechanism similar to that originating saddle splay layer distortion in the so-called helical nanofilament (HNF) phases of BCMs [7,8,46,47].

## Figures and Tables

**Table 1:** Results of fitting the NR curves: fitted parameters, best fit values, 65% confidence interval, and limits of variation. The background parameters indicate the amount of uncoherent scattering from the sample. Reduced *χ*^2^ = 3.1667.

Parameter	Fitted Value	65% Confidence Interval	Limits (Min-Max)
Layer 1 thickness/Å	27.8	23.1, 32.3	1–50
Layer 2 thickness/Å	24.8	22.5, 31.1	1–50
Layer 1 *APM*/Å^2^	30.5	28.5, 37.1	1–50
Layer 2 *APM*/Å^2^	77.5	57.4, 110	1–150
Layer 2 hydration/%	77.0	53.4, 78.8	0–100
Background D_2_O/×10^−6^	1.65	0.890, 7.70	0.1–100
Background NRW/×10^−6^	6.15	3.30, 7.24	0.1–100

## Data Availability

The data presented in this study are available upon request from the corresponding author.
